# Enhanced Intestinal Absorption of Insulin by Capryol 90, a Novel Absorption Enhancer in Rats: Implications in Oral Insulin Delivery

**DOI:** 10.3390/pharmaceutics12050462

**Published:** 2020-05-18

**Authors:** Hiroki Ukai, Kazuki Iwasa, Takamasa Deguchi, Masaki Morishita, Hidemasa Katsumi, Akira Yamamoto

**Affiliations:** Department of Biopharmaceutics, Kyoto Pharmaceutical University, Misasagi, Yamashina-Ku, Kyoto 607-8414, Japan; kd17003@ms.kyoto-phu.ac.jp (H.U.); baseba11iwasa@gmail.com (K.I.); tkms.exit@gmail.com (T.D.); morishita@mb.kyoto-phu.ac.jp (M.M.); hkatsumi@mb.kyoto-phu.ac.jp (H.K.)

**Keywords:** drug absorption, absorption enhancer, insulin, intestinal absorption, peptide absorption, Caco-2 cells, tight junction

## Abstract

Labrasol^®^ is a self-emulsifying excipient that contains saturated polyglycolysed C_6_–C_14_ glycerides and this additive is known to improve the intestinal absorption of poorly absorbed drugs after oral administration. However, the effects of formulations similar to Labrasol^®^ on the intestinal absorption of poorly absorbed drugs have not been characterized. In this study, we used insulin as a model peptide drug and examined the absorption-enhancing effects of Labrasol^®^ and its related formulations for insulin absorption in rats. The co-administration of Labrasol-related formulations with insulin reduced the blood glucose levels. Among these formulations, Capryol 90 was the most effective additive. Notably, the effect of Capryol 90 was greater at pH 3.0 than at pH 7.0. Additionally, almost no mucosal damage was observed in the presence of these formulations, as these formulations did not affect the activity of lactate dehydrogenase (LDH) and the amount of protein released from the small intestine. In mechanistic studies, Capryol 90 improved the stability of insulin and suppressed the association with insulin under acidic conditions. The loosening of the tight junctions (TJs) could be the underlying mechanism by which Capryol 90 improved intestinal insulin absorption via a paracellular route. These findings suggest that Capryol 90 is an effective absorption enhancer for improving the intestinal absorption of insulin, without inducing serious damage to the intestinal epithelium.

## 1. Introduction

As medical technology advances and several therapeutic agents are being developed, the demand for easy-to-administer and non-invasive formulations also increases. Oral administration is a promising administration method resulting in improved patient compliance, and thus is the most widely used route in current drug therapy. However, to enhance the absorption of target drugs from the gastrointestinal (GI) tract, many obstacles need to be overcome. In particular, peptide and protein drugs are generally administered by injectable formulations, since peptidases and proteases can promote the degradation of these drugs in the GI tract [1].

Various strategies have been studied to improve the intestinal absorption of peptide and protein drugs. Among these strategies, the use of formulation additives, including absorption enhancers and protease inhibitors, remains a promising approach [2,3,4,5,6,7]. However, few absorption enhancers have been utilized in practical applications. For example, sodium caprate, a typical fatty acid, has been employed as an absorption enhancer in suppositories of ampicillin or ceftizoxime, and its efficacy and toxicity have been documented [8]. More recently, the most advanced enhancers in clinical trials for oral peptide delivery comprise a medium-chain fatty acid-derived salt: sodium *N*-[8-(2-hydroxybenzoyl)amino]caprylate (SNAC) [9]. Conversely, Labrasol^®^ is a self-emulsifying non-ionic surfactant, and its absorption-enhancing effects on the GI tract have been previously reported [10,11,12]. Labrasol^®^ conforms to the caprylocaproyl macrogol-8 glycerides and the caprylocaproyl polyoxyl-8 glycerides. It is composed of mono-, di-, and triglycerides, as well as mono- and di- fatty acid esters of polyethylene glycol (PEG) and free PEG-8 [13,14]. One of the advantages of the self-microemulsion system is that the mixture of the drug and emulsifier enables the formation of fine microemulsions, with only gentle immediate agitation after the formulation blends with the intestinal fluid. Therefore, the efficacy of Labrasol^®^ for poorly absorbable drugs has been actively studied. Notably, Labrasol^®^ increases the intestinal absorption of gentamicin and insulin [10,12]. Other studies indicate that this excipient increases the bioavailability of low-molecular-weight heparin following rat jejunal instillations [15]. Furthermore, we have assessed the intestinal absorption of carboxyfluorescein (CF) and FITC-dextran 4000 (FD4), and 10% (*v*/*v*) Labrasol^®^ increases the bioavailability of these compounds [16]. To mediate this action of Labrasol^®^, C_8_ and C_10_ moieties are important components, as well as PEG esters and glycerides [17]. In addition, it has been clarified that the diethyl ether fraction of Labrasol^®^ exhibits a strong absorption-enhancing effect [11]. Thus, considerable attention has been focused on the effects of Labrasol^®^. However, the absorption-enhancing effects of Labrasol-related formulations need to be comprehensively elucidated.

Here, we first examined the effects of Labrasol^®^ and its related formulations on the intestinal absorption of the peptide drug, insulin. In this study, we used Labrasol^®^, Capryol 90, Capryol PGMC, Lauroglycol 90, Lauroglycol FCC, Labrafil M 2125 CS, and Maisine 35-1. The compositions of each formulation are listed in Table 1. Secondly, intestinal membrane damage in the presence of these formulations was estimated by measuring the activity of lactate dehydrogenase (LDH) and the amount of protein released from intestinal membranes in rats. Thirdly, we performed mechanistic studies to clarify the possible mechanisms underlying the enhancing effects of these excipients on the intestinal absorption of insulin.

## 2. Materials and Methods 

### 2.1. Materials

Male Wistar rats (220–270 g, 8 weeks of age) were obtained from SLC, Inc. (Hamamatsu, Shizuoka, Japan). The Glucose C2 Test, LDH Cytotoxicity Test and recombinant human insulin were purchased from Wako Pure Chemical Industries, Ltd. (Osaka, Japan). Labrasol^®^, Capryol 90, Capryol PGMC, Lauroglycol 90, Lauroglycol FCC, Labrafil M 2125 CS, and Maisine 35-1 were obtained from Gattefossé (Saint-Priest, France). Hydrogenated castor oil-60 (HCO-60) was obtained from Nikko Chemical Co., Ltd. (Osaka, Japan). Triton X-100, high glucose Dulbecco’s modified Eagle’s medium (DMEM), MEM non-essential amino acid solution (MEM-NEAA), and antibiotic–antimycotic mixed stock solution (10,000 IU/mL penicillin, 10 mg/mL streptomycin, and 25 μg/mL amphotericin B) were purchased from Nacalai Tesque, Inc. (Kyoto, Japan). Fetal bovine serum (FBS) was obtained from Equitech-Bio, Inc. (Kerrville, TX, USA). Hank’s balanced salt (HBS) was obtained from Sigma-Aldrich Chemical Co. (St. Louis, MO, USA). 2-[4-(2-Hydroxyethyl)-1-piperazinyl] ethanesulfonic acid (HEPES) was prepared by Dojindo Laboratories (Kumamoto, Japan). Caco-2 cells were bought from Dainippon Sumitomo Pharma Co., Ltd. (Osaka, Japan). 

### 2.2. Intestinal Absorption of Insulin Using the In Situ Closed-Loop Method

The intestinal absorption of insulin was examined using an in situ closed-loop method in rats, as previously reported [6]. The experiments were performed in accordance with the guidelines of the Animal Ethics Committee of the Kyoto Pharmaceutical University (Permit number; 17-021). The number of rats used in this experiment was three to four in each group. As for the dosing solutions, phosphate-buffered saline (PBS, pH 7.0 or 3.0) containing 1% (*w*/*v*) HCO-60 was prepared and Labrasol^®^, Capryol 90, Capryol PGMC, Lauroglycol 90, Lauroglycol FCC, Labrafil, or Maisine were added to the dosing solutions at concentrations of 10% (*v*/*v*). We also prepared three different concentrations (5%, 10%, and 15% *v*/*v*) of dosing solutions in the case of Capryol 90. Specific amounts of recombinant human insulin were dissolved in 0.01 M HCl. The insulin solution was mixed with absorption enhancers in PBS solution. The rats were fasted overnight for 14 h before the experiment, but had free access to water. Following intraperitoneal injection of sodium pentobarbital at a dose of 32 mg/kg body weight, the anesthetized rats were placed under a heating lamp and their body temperatures were maintained on a heating plate at about 37 °C. A midline abdominal incision was used to expose the small or large intestines and these intestines were rinsed with pH 7.4 PBS and air. After ligating the bile duct, the intestine was cannulated at both proximal and distal parts with polyethylene cannulas (internal diameter, 3 mm; outer diameter, 5 mm) clipped by forceps. The dosing solutions (3 mL for the small intestine and 1 mL for the large intestine), warmed to 37 °C, were directly injected into the intestinal loops at a dose of 80 IU/kg body weight, through the cannula at the proximal part, which was then closed during the absorption study. The insulin solution without the addition of the formulation was used as control group in this experiment. The jugular vein was exposed and 0.2 mL of blood was collected using heparinized syringes at predetermined time points up to 240 min. The blood samples were immediately separated from the plasma fraction by centrifuging the samples at 12,000 rpm (9660× *g*) for 5 min, and plasma fractions were stored on ice until assay. The method for determining the plasma glucose concentrations in these samples was described below.

In the pretreatment experiment, Capryol 90 (10%, *v*/*v*) was administered into the small intestinal loop. After pretreatment with Capryol 90 for 60 min, the Capryol 90 solution was removed by washing the intestinal lumen with PBS (pH 7.4). Immediately after washing, the insulin solution was administered into the small intestinal loop, and plasma samples were collected to determine the glucose concentrations.

The pharmacological availability (PA%) was calculated using data obtained by intravenous administration of insulin solution (1 IU/kg). 

### 2.3. Estimation of Intestinal Membrane Damage

The activity of LDH and the amount of protein released from intestinal epithelial cells were examined to assess the intestinal toxicity of Labrasol^®^ and its related formulations [4,6]. Solution with or without 10% (*v*/*v*) of each excipient or 3% (*v*/*v*) Triton X-100, used as a positive control, was administered into the intestinal loops of experimental animals in the same manner as that used for the in situ intestinal absorption experiments. After 4 h of treatment, the intestine was washed with 30 mL of PBS (pH 7.4) and the washing solution was collected to determine the damage to the intestinal membrane. The supernatant of the samples was obtained by centrifuging these samples for 7 min at 200× *g* at 4 °C, and the membrane damage was estimated by measuring two toxicological markers in the supernatant samples. LDH activity and protein amount were determined using the LDH Cytotoxicity Test kit and Bradford method, respectively. The measurement was performed with a microplate multi-detection reader (Synergy HT with Gen5 Software; BioTek Instruments, Inc., Winooski, VT, USA).

### 2.4. Circular Dichroism Studies

A circular dichroism (CD) spectrophotometer (J-1500-450STG, JASCO Co., Tokyo, Japan) was utilized to scan from 300 to 250 nm, at a rate of 50 nm/min at 25 °C. Human insulin was dissolved in PBS (pH 7.0 or 3.0) at a concentration of 0.02 mM, and 0.5% (*v*/*v*) Capryol 90 was added to the insulin solution. Each solution was analyzed in a 1-cm pathlength quartz cuvette. Three consecutive scans were averaged for each sample. The molar ellipticity was calculated using the following equation:

[θ]_λ_ = θ_λ_/(*C* × *l*) × 100

where θ_λ_ is the observed ellipticity at a wavelength λ in degrees, *C* is the molar insulin concentration, and *l* is the path length in centimeters.

### 2.5. Preparation of Rat Mucosal Tissue Homogenates

Mucosal tissue homogenates of the rat small intestine were prepared using the modified method previously reported by Yamamoto et al [18]. The rats were fasted overnight and euthanized. After washing the luminal surface with PBS (pH 7.4), the small intestine was removed, and the mucosal tissue was scraped using a glass plate. The specimens were homogenized in 1–3 mL of PBS at 4 °C using a Polytron homogenizer (Kinematica, GmbH, Lucerne, Switzerland). The homogenates were separated by centrifugation (8000× *g*, 4 °C) for 5 min. The supernatant samples were diluted with PBS to obtain a protein concentration of 10 mg/mL.

### 2.6. Degradation of Insulin in Intestinal Mucosal Homogenates

The degradation of insulin was evaluated by incubating 240 µL of mucosal tissue supernatant with 360 µL of 0.02 mM insulin solution, including Capryol 90 (0.5% *v*/*v*) and HCO-60 (0.1% *w*/*v*) as solubilizing agents. At predetermined time points up to 240 min, samples were withdrawn from the incubation mixtures. After adding the same volume of acetonitrile to terminate the reaction, the samples were immediately centrifuged twice at 9000× *g* at 4 °C for 5 min to remove the precipitated protein. The remaining insulin in the supernatant was determined by high-performance liquid chromatography (HPLC).

### 2.7. Measurement of Transepithelial Electrical Resistance (TEER) and Transport of Insulin Using Caco-2 Cell Monolayers

Caco-2 cells were cultured in 175 cm^2^ culture flasks (Thermo Fisher Scientific^TM^, Waltham, MA, USA). The culture medium consisted of DMEM supplemented with 10% FBS, 1% MEM-NEAA and 1% antibiotic–antimycotic mixed stock solution. Caco-2 cells were cultured in a humidified atmosphere containing 5% CO_2_ at 37 °C. When the cultured Caco-2 cells became sub-confluent, Caco-2 cells were seeded onto Polycarbonate Membrane Transwell^®^ inserts (pore size: 0.4 µm, growth surface area: 1.12 cm^2^ and membrane diameter: 12 mm, Corning Inc., Corning, NY, USA) at 2.0 × 10^5^ cells/mL. The integrity of Caco-2 cells was monitored by measuring TEER values using a Millicell^®^ ERS-2 Volt-Ohm Meter (Millipore Corporation, Burlington, MA, USA), and the medium was changed every 2 days. Transepithelial transport studies were performed when the TEER values were more than 500 Ω·cm^2^ (i.e., after 21–23 days). After removing the incubation medium by aspiration, the cells were incubated with modified HBS solution (mHBSS, containing 10 mM HEPES) (pH 6.5) at the apical side and mHBSS (pH 7.4) at the basal side for 60 min at 37 °C. Following preincubation, 0.5 mL of mHBSS (pH 6.5) containing insulin (0.04 mM), with or without Capryol 90 was added to the apical side at the zero-time point, whereas precisely 1.5 mL of mHBSS (pH 7.4) was added to the basal side. At predetermined time points up to 360 min, TEER was measured, followed by sampling 150 μL aliquots from the basal side, and 150 μL of insulin-free mHBSS, which was pre-heated to 37 °C, was added immediately. Each sample was mixed with an equal volume of acetonitrile and was centrifuged (9000× *g*, 4 °C) for 5 min. The transport of insulin from the apical compartment to the basolateral compartment was measured by HPLC. The apparent permeability coefficients (*P*_app_) for the transported insulin were determined using the following equation:

*P*_app_ = *Flux*/(*A* × *C*_0_ × 60),

where *P*_app_ is the apparent permeability coefficient (cm/s), *Flux* is the slope of the linear portion of the cumulative transport profile at the last three points (nmol/min), *A* is the membrane surface area (1.12 cm^2^), and *C*_0_ is the initial concentration of the insulin in the donor side (nmol/mL).

### 2.8. Analytical Methods

The intestinal absorption of insulin was estimated by insulin-induced hypoglycemia. Using the Glucose C2 Test Kit, we determined the glucose concentrations in plasma. We also calculated the area above the curve (AAC) using the trapezoidal method, from time zero to the final sampling time. 

*PA*% was obtained using the following equation:

*PA*% = (*AAC*_G.I._/*AAC*_i.v._) × (*Dose*_i.v._/*Dose*_G.I._) × 100

where *AAC*_G.I._ is the AAC value following insulin administration to the intestinal loop, *AAC*_i.v._ is the AAC value following intravenous insulin administration, *Dose*_i.v._ is the insulin dose after intravenous administration (1 IU/kg), and *Dose*_G.I._ is the insulin dose after administration to the intestinal loop (80 IU/kg).

The stability and transport of insulin were determined by HPLC. The HPLC system consisted of a (LC-20AD; Shimadzu, Kyoto, Japan) pump equipped with an ultraviolet detector (210 nm). The mobile phase was composed of 0.1% trifluoroacetic acid (TFA) in water (mobile phase A) and 0.1% TFA in acetonitrile (mobile phase B). Each mobile phase was mixed by the gradient program, i.e., mobile phase A and B mixed at 75:25 and changed gradually to 60:40 over 15 min, then held at 75:25 for 5 min. The mobile phase was injected in a separation column (COSMOSIL 5C18-AR300; 4.6 × 150 mm, Nacalai Tesque Inc., Kyoto, Japan) at a flow rate of 1.0 mL/min.

### 2.9. Statistical Analyses

Each value represents the mean ± standard error (S.E.) of at least three experiments. For multiple comparisons, statistical difference was calculated using the Tukey–Kramer test. Differences were considered significant at *p* < 0.05.

## 3. Results

### 3.1. Effects of Labrasol^®^ and Its Related Formulations on the Intestinal Absorption of Insulin and Their Intestinal Membrane Toxicity

**Figure 1:**
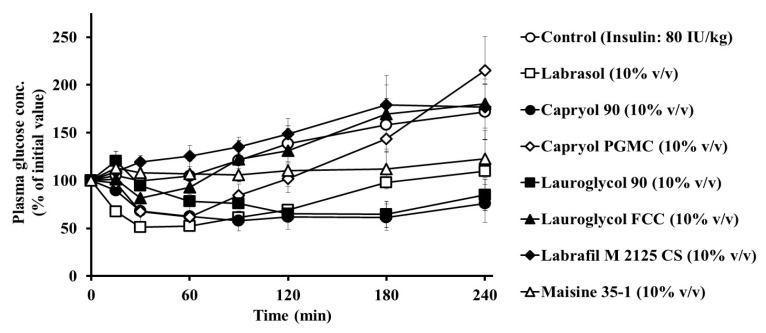
The effects of Labrasol^®^ and its related formulations on plasma concentrations of glucose after small intestinal administration of insulin (80 IU/kg) using an in situ closed loop. Glucose concentrations are expressed as a percentage of the initial concentration at time zero. Each value represents the mean ± standard error (S.E.) of three to four rats.

Figure 1 shows effects of Labrasol^®^ and its related formulations on the intestinal insulin absorption. The blood glucose levels at the start of the experiments were around 100 mg/dL (5.5 mM) in all tested rats. Following the intestinal administration of insulin alone, almost no effect was observed on the plasma glucose levels, with significant hypoglycemic effects observed following the intestinal administration of Labrasol^®^, Capryol 90, and Lauroglycol 90 in the insulin solution when compared with the control group. Table 2 summarizes the pharmacodynamic parameters following insulin administration with each absorption enhancer to the small intestinal loops of rats. As indicated in Table 2, the AAC values and PA% of insulin in the absence of absorption enhancers were markedly low. In contrast, Labrasol^®^ and its related formulations enhanced the intestinal absorption of insulin, and the PA% values of insulin increased with Labrasol^®^, Capryol 90, and Lauroglycol 90 by nine-, 13-, and nine-fold, respectively. Furthermore, the effects of Capryol 90 on the small intestinal absorption of insulin were evaluated at three different concentrations (5%, 10%, and 15% *v*/*v*). Capryol 90 increased the intestinal absorption of insulin in a concentration-dependent manner (Table 2). These results indicated that Labrasol^®^ Capryol 90, and Lauroglycol 90 evaluated in this study enhanced the intestinal absorption of insulin. Among these additives, Capryol 90 was the most effective absorption enhancer. 

**Figure 2:**
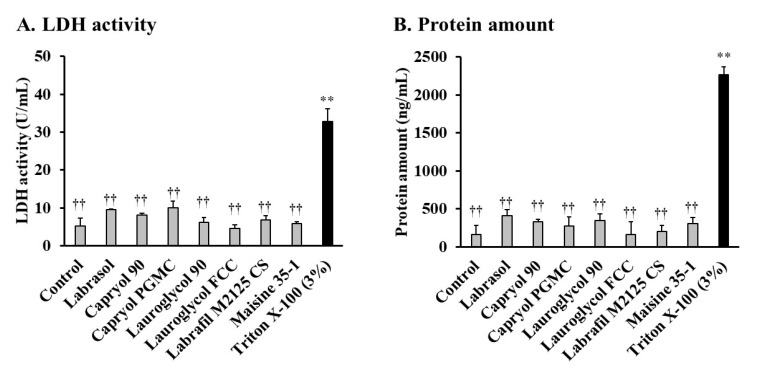
Effects of 10% (*v*/*v*) Labrasol^®^ and its related formulations on the (**A**) lactate dehydrogenase (LDH) activity and (**B**) amount of protein released from rat small intestinal mucosa by an in situ closed-loop study. Each value represents the mean ± S.E. of at least three experiments. (**) *p* < 0.01, compared with the control group. (††) *p* < 0.01, compared with the positive control group.

To estimate the safety of the various enhancers investigated in this study, the activities of LDH and the amount of protein released from the small intestine were evaluated. As shown in Figure 2, at a concentration of 10%, none of the enhancers increased the LDH activity. In the presence of these enhancers, LDH activity was considerably lower than that with 3% Triton X-100, used as a positive control (Figure 2A). Similar results were also observed in terms of the amount of protein released with these absorption enhancers (Figure 2B). These results indicated that Labrasol^®^ and its related formulations did not induce serious membrane damage to the small intestinal membrane. Based on the effectiveness and toxicity studies, Capryol 90 was selected as a suitable absorption enhancer in subsequent studies.

### 3.2. Absorption Enhancing Characteristics of Capryol 90

**Figure 3:**
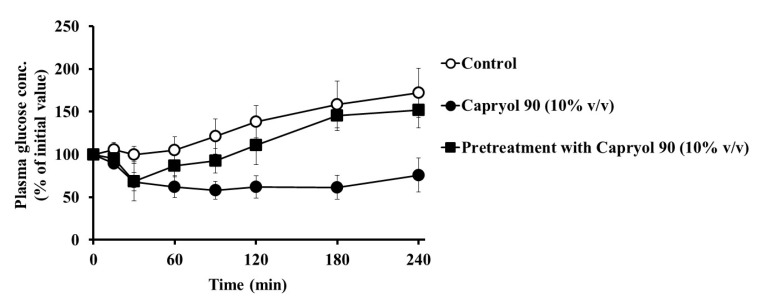
Effect of 10% (*v*/*v*) Capryol 90 pretreatment on the absorption of insulin from the small intestine using an in situ closed loop. Each value represents the mean ± S.E. (*n* = 3).

To determine whether the effects of Capryol 90 on intestinal insulin absorption were reversible, the effect of 10% Capryol 90 pretreatment on insulin absorption was examined. As shown in Figure 3, Capryol 90 markedly reduced the plasma glucose concentration when simultaneously administered with insulin (80 IU/kg). In contrast, a slight decrease in the blood glucose concentration was observed following pretreatment with Capryol 90 for 60 min and the administration of insulin. The hypoglycemic effect of insulin was observed during the initial 30 min in the pretreatment group, but this effect quickly disappeared and the blood level increased at later time points. However, the result was not significant when compared with the control. The *AAC*_0__→4h_ was 8050 ± 703%·min (** *p* < 0.01) in the presence of 10% Capryol 90, whereas the *AAC*_0__→4h_ was 2520 ± 1300%·min (not significant) following pretreatment with 10% Capryol 90. These results indicate that the removal of Capryol 90 from the rat small intestine reduced the absorption-enhancing effect of Capryol 90, suggesting that this effect was reversible. Therefore, Capryol 90 may not cause continuous and irreversible damage to the intestinal membrane.

**Figure 4:**
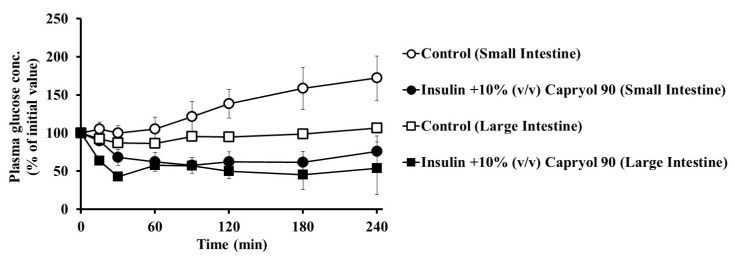
Regional difference in the effect of Capryol 90 on the intestinal absorption of insulin using an in situ closed loop. Insulin absorption following an 80 IU/kg dose was estimated by measuring plasma glucose levels. Each value represents the mean ± S.E. (*n* = 3).

Furthermore, we studied the absorption-enhancing effect of Capryol 90 on insulin absorption in the large intestine and compared the results with those in the small intestine group. As shown in Figure 4, we observed a hypoglycemic effect (** *p* < 0.01) after the co-administration of insulin with Capryol 90 (10%, *v*/*v*) in the large intestine when compared with the control group. Following administration in the large intestine, the *AAC*_0__→4h_ value of the control group was 1370 ± 250%·min (*PA*% = 0.21 ± 0.04), whereas the *AAC*_0__→4h_ was 11,490 ± 2150%·min (*PA*% = 1.76 ± 0.33; ** *p* < 0.01) in the presence of 10% Capryol 90. The decrease in the plasma glucose levels was similar to that in the small intestinal administration group. Therefore, it was revealed that Capryol 90 significantly enhances insulin absorption in both the small and large intestines.

**Figure 5:**
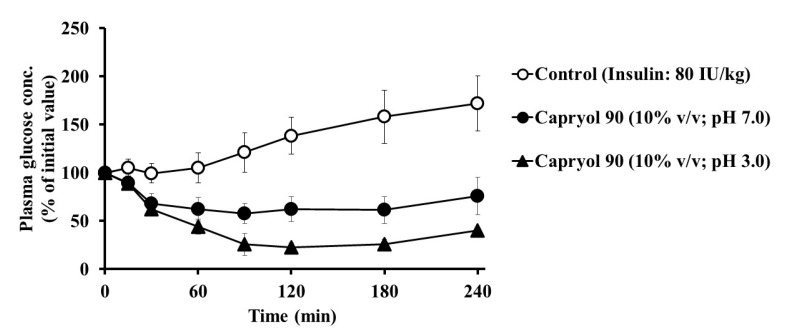
Effect of insulin solution pH on the intestinal absorption of insulin with Capryol 90. Each value represents the mean ± S.E. of three experiments.

In general, the activity of proteolytic enzymes in the intestine varies with the pH in the digestive tract. Additionally, the conformation of insulin is affected by the solution pH, and therefore intestinal insulin absorption may be altered under different pH environments. We studied the absorption-enhancing effect of Capryol 90 on insulin in a low pH condition of the dosing solution. As shown in Figure 5, we observed hypoglycemic effects following the co-administration of insulin with Capryol 90 (10%, *v*/*v*) in a low pH solution (pH 3.0). The decrease in the plasma glucose levels at pH 3.0 was greater than observed in the pH 7.0 solution, with AAC values increased from 8050 ± 703%·min (*PA*% = 1.23 ± 0.11) to 14,700 ± 917%·min (*PA*% = 2.25 ± 0.14). These findings suggest that the absorption-enhancing effect of Capryol 90 on insulin absorption is increased under low pH (pH 3.0) conditions when compared with that in a dosing solution at pH 7.0.

### 3.3. Effect of Capryol 90 on the Association Properties of Insulin 

**Figure 6:**
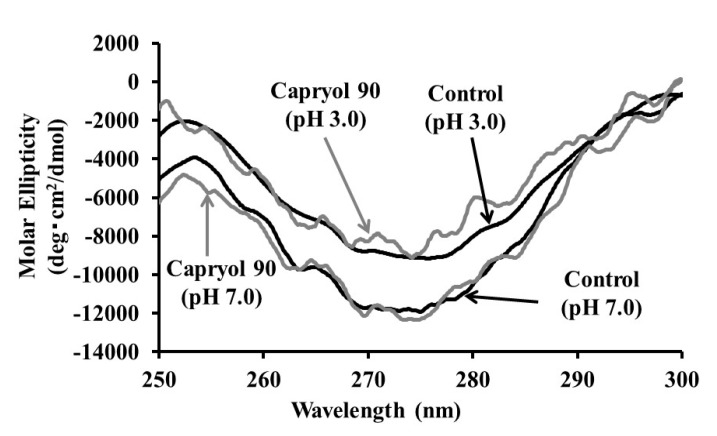
Effects of Capryol 90 and pH on circular dichroic spectra of 0.02 mM insulin.

It is well known that insulin exists in monomers, dimers, tetramers, and hexamers. Therefore, the effect of Capryol 90 on insulin association was investigated using the CD spectra. Decreased molar ellipticity at 270 nm indicated the association of insulin in the solution. As shown in Figure 6, in the CD spectra of insulin at pH 3.0, the negative maximum at 270 nm was attenuated when compared with insulin at pH 7.0. In contrast, Capryol 90 failed to significantly attenuate the negative maximum value for molar ellipticity when compared with that of insulin alone. These results demonstrated that the association property of insulin was only slightly affected by Capryol 90, but was remarkably suppressed by low pH conditions.

### 3.4. Effect of Capryol 90 on the Stability of Insulin in Intestinal Homogenates 

**Figure 7:**
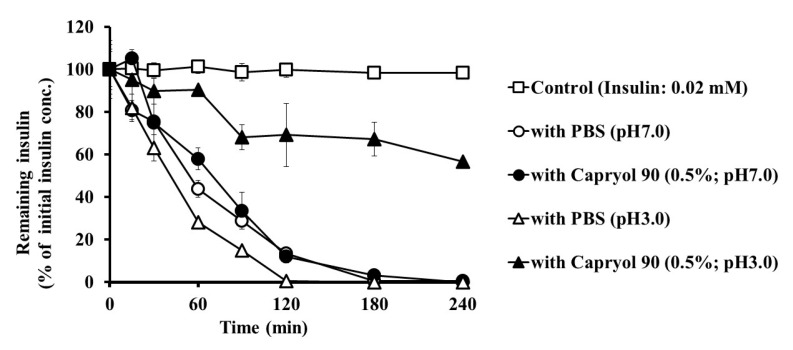
Effects of Capryol 90 and pH on the degradation of insulin in small intestine homogenates at 37 °C. Each value represents the mean ± S.E. (*n* = 3).

To elucidate the absorption-enhancing mechanisms of Capryol 90, we examined the effect of Capryol 90 on insulin degradation in the small intestinal mucosa. The insulin solution and PBS, with or without Capryol 90 (pH 7.0 or 3.0), were mixed and added to the rat small intestinal mucosal homogenate solutions, and the remaining insulin content was measured during the incubation period (Figure 7). Additionally, insulin dissolved in PBS without the intestinal homogenate was used as a control. In rat small intestinal homogenates, insulin was rapidly degraded to less than 10% of the starting concentration at 120 min. In the pH 7.0 solution, insulin was degraded similarly to when Capryol 90 was added in the homogenates. In contrast, more than 50% of insulin remained after 120 min with Capryol 90 in the pH 3.0 solution. The half-life values of insulin degradation were 61.9 ± 8.8 and 64.8 ± 11.0 in pH 7.0, and 41.1 ± 5.3 and 150.2 ± 19.1 in pH 3.0 solutions, with or without Capryol 90, respectively. In the pH 3.0 solution, insulin was markedly degraded without Capryol 90. Therefore, it was suggested that Capryol 90 was effective in reducing the degradation of insulin under low pH conditions.

### 3.5. Effects of Capryol 90 on TEER and Insulin Transport in Caco-2 Cell Monolayers 

**Figure 8:**
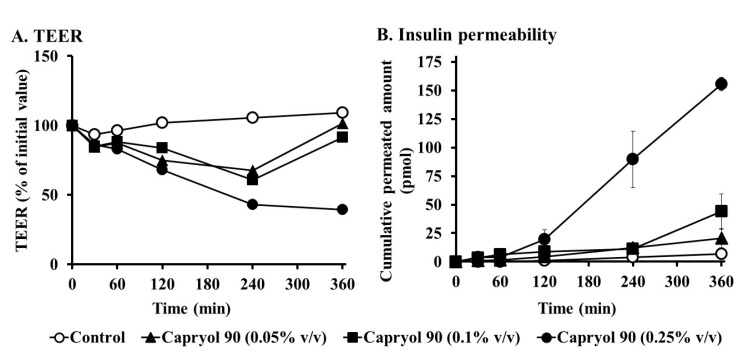
Effect of Capryol 90 with different concentrations on transepithelial electrical resistance (TEER) and permeation of insulin through Caco-2 cell monolayers. (**A**) TEER values (TEER % of initial value). (**B**) Cumulative permeated amount of insulin in the presence of Capryol 90. Each value represents the mean ± S.E. (*n* = 3).

Next, we examined whether Capryol 90 enhances the intestinal absorption of insulin via a paracellular route. Capryol 90 at concentrations of 0.05% (*v*/*v*), 0.1% (*v*/*v*), and 0.25% (*v*/*v*) were added to the apical side at 37 °C. As shown in Figure 8A, treatment with all concentrations of Capryol 90 reduced TEER values (represented as a percentage of the initial value) of Caco-2 cells. Capryol 90 at concentrations of 0.05% and 0.1% decreased the TEER values during the initial 240 min, with recovery to baseline observed at later time points. At a concentration of 0.25%, Capryol 90 decreased TEER values to a greater extent than that observed at concentrations of 0.05% and 0.1%. Furthermore, we also examined the effects of Capryol 90 on the permeability of insulin across Caco-2 cell monolayers. As shown in Figure 8B, all concentrations of Capryol 90 increased the permeability of insulin. In the control group, the *P*_app_ value of insulin was 0.24 ± 0.1 × 10^−7^ (cm/s), whereas 0.05%, 0.1% and 0.25% Capryol 90 increased the *P*_app_ of insulin to 0.68 ± 0.3 × 10^−7^ (cm/s), 1.45 ± 0.6 × 10^−7^ (cm/s), and 5.67 ± 0.6 × 10^−7^ (cm/s), respectively. These results suggest that Capryol 90 might loosen tight junctions (TJs) in the intestinal epithelium, thereby increasing intestinal insulin transport via the paracellular pathway.

## 4. Discussion

The present study demonstrates that Labrasol^®^ and its related formulations can enhance the intestinal insulin absorption from the intestine. In the present study, we added HCO-60 to maintain the solubility of the tested additives. Previously, we reported that the intestinal absorption of insulin was not changed by the addition of HCO-60 [16,19]. In addition, all dosing solutions, including the control group, contain HCO-60 in this study. Therefore, it is considered that we can rule out the absorption-enhancing effect of HCO-60 for the intestinal absorption of insulin in this study. 

We observed that Labrasol^®^ and its related formulations enhanced the intestinal absorption of insulin, as shown in Figure 1. Depending on the AAC and PA% values of insulin, the rank order of the effectiveness of absorption enhancers in the small intestine was as follows: Capryol 90 > Labrasol^®^ = Lauroglycol 90 > Capryol PGMC > Maisine > Lauroglycol FCC > Labrafil, as shown in Table 2. It is not fully understood why differences exist among the formulations in terms of their effectiveness in enhancing the intestinal insulin absorption. However, it appears that the absorption efficacy is related to the length of the carbon chain of fatty acids. It is well known that medium-chain fatty acids are extremely effective in causing turbulence in the intestinal membrane and TJs located between the intestinal epithelial cells [20]. This feature is also common to the latest technologies such as GIPET^®^, which is undergoing clinical research [21,22]. In particular, Capryol 90 contains 90% of the monoester of caprylic acid (eight carbon atoms), higher than that present in other formulations. We believe that the high content of the caprylic acid monoester is one of the factors responsible for the enhanced effect of Capryol 90 on the absorption of insulin when compared to other formulations. Regarding its superior effect in the preliminary studies, Capryol 90 was chosen from among the other formulations to conduct additional studies and further elucidate its specific actions and mechanisms.

When absorption enhancers are applied clinically, their local irritation and toxicity to the intestinal membrane should be considered and evaluated. In this study, we used the activities of LDH as one of the toxicological markers, since an increase in the LDH activity in the luminal fluid is usually considered evidence of cellular membrane damage. We observed that both Labrasol^®^ and its related formulations did not increase LDH activity in the small intestine (Figure 2A). Additionally, proteins are components of biological membranes, released when biomembranes are damaged. Hence, they are generally considered an index of membrane damage. As shown in Figure 2B, we measured the protein release from intestinal epithelial cells. Labrasol^®^ and its related formulations did not increase the protein release from intestinal epithelial cells. These results suggest that these additives are not toxic to the intestinal membranes.

Our study showed that Capryol 90 enhanced intestinal insulin absorption in a concentration-dependent manner (Figure 1 and Table 2). We also evaluated the reversibility of the absorption-enhancing effect of 10% Capryol 90 (Figure 3). Pretreatment of the small intestine with 10% (*v*/*v*) Capryol 90 had negligible effects on plasma glucose levels. However, co-administration of Capryol 90 enhanced the insulin absorption in the small intestine. Therefore, the absorption-enhancing effect of Capryol 90 was reversible, supporting the safety of Capryol 90.

Furthermore, regional differences were observed in the enhancement induced by Capryol 90 on the intestinal insulin absorption. However, this regional difference was not markedly pronounced, as shown in Figure 4. Capryol 90 significantly enhanced the absorption of insulin from the small and large intestine. Additionally, insulin itself is better absorbed from the large intestine than from the small intestine. In general, the large intestine shows lower proteolytic enzyme activity, which can partly explain the higher pharmacological action of insulin in the large intestine when co-administered with Capryol 90. Previously, we have reported that the absorption-enhancing effects of capric acid and its related compounds were greater in the large intestine than in the small intestine [19]. In contrast, Numata et al. reported that 1-hydroxy-2-oxo-3-(*N*-methyl-3-aminopropyl)-3-methyl-1-triazene (NOC7), a nitric oxide donor, significantly improved the intestinal absorption of FD4 in every intestinal region [23]. Moreover, we reported that the intestinal absorption of insulin and [Asu^1,7^]-eel calcitonin was enhanced in all intestinal regions [3]. These findings are in good agreement with the previous data of NO donors applied to the different intestinal regions. We also indicate that Capryol 90 could increase the small and large intestinal absorption of poorly absorbed drugs, although the absorption-enhancing effects of conventional absorption enhancers are usually greater in the large intestine than in the small intestine.

Interestingly, the effect of Capryol 90 on the absorption of insulin was more pronounced under low pH conditions than under neutral conditions, as shown in Figure 5. Considering the implications of oral insulin delivery, this phenomenon is extremely significant, indicating that Capryol 90 may perform better than expected during oral administration through the stomach, which is an acidic environment. Generally, the activity of proteolytic enzymes and the association of insulin are affected by the pH conditions [24,25]. We hypothesized that these factors may influence the insulin absorption-enhancing effect of Capryol 90.

Furthermore, we examined and elucidated the absorption-enhancing mechanisms of Capryol 90, the superior additive in this study. We measured the CD spectra to confirm the effect of Capryol 90 on the association of insulin, since the conformation of insulin can be evaluated by the CD spectra. We estimated the insulin association with the antiparallel beta structure by the CD spectra—namely, if the phenylalanine and tyrosine residues in the B_23–28_ region have antiparallel beta structure, we can observe negative CD absorption at 270 nm. Therefore, attenuation of the negative maximum at 270 nm indicates the dissociation of insulin aggregates [26,27]. As indicated in Figure 6, the addition of Capryol 90 slightly changed the CD spectrum at 270 nm of insulin. In contrast, we observed remarkable attenuation of the CD spectrum of insulin when the pH of the solution was lowered from 7.0 to 3.0. These results suggest that insulin association was inhibited under acidic conditions, while Capryol 90 itself demonstrated a minimal effect on insulin association. The insulin association equilibrium is influenced by divalent cations, the solution pH, and salt type [28,29,30,31]. Furthermore, the amino acid residues responsible for the oligomerization of insulin have been identified [32]. In particular, it has been reported that acid stabilization of the insulin conformation involves protonation of histidine at position 5 on the B-chain (HB5) [30].

Moreover, we observed that Capryol 90 marginally prevented insulin degradation in small intestine homogenates in the neutral pH solution, as shown in Figure 7. In contrast, in a low pH (3.0) solution, Capryol 90 improved the stability of insulin. Furthermore, acidic conditions without Capryol 90 did not improve insulin stability. Hence, Capryol 90 itself may have a protective effect on insulin degradation or may inactivate digestive enzymes in low pH solutions. As shown in Figure 6, insulin dissociates under low pH conditions, which may alter the insulin properties. We postulated that a lower apparent molecular weight would result in reduced stability, owing to an attack by digestive enzymes. However, the opposite result was observed. As mentioned above, as the antiparallel beta structure, phenylalanine, and tyrosine residues in the B_23–28_ region, are formed by insulin association, the dissociation of insulin aggregates may expose these aromatic amino acid residues. This phenomenon increases the solubility of insulin in lipids, increasing the likelihood of encapsulation within particles composed of Capryol 90. Therefore, Capryol 90 protects insulin and increases the stability of insulin in low pH conditions. Considering the nanosystem which has been frequently reported in recent years, the results presented in this study may provide a simpler absorption enhancing technology [33]. The use of complex technology, such as nanocarriers, liposomes, etc., is highly effective [34,35,36,37]. While these require complicated techniques, we found that Capryol 90 improved insulin stability and intestinal absorption simply by mixing it into the formulation. Thus, we think that Capryol 90 is excellent due to its convenience and low cost. However, further studies are required to understand the impact of the relationship between Capryol 90 and solution pH on insulin association, stability, and intestinal absorption. Conversely, we observed the absorption-enhancing effect of Capryol 90 on insulin in the neutral dosing solution, as shown in Figure 1. Therefore, a stabilizing effect on insulin may not be essential for Capryol 90 to exert its absorption-enhancing effect. 

In this study, we used Caco-2 cell monolayers to elucidate the absorption enhancing mechanisms of Capryol 90. In this study, lower concentrations of Capryol 90 (0.05%, 0.1%, and 0.25%), were used for the transport studies of Caco-2 monolayers, since these cells were generally very sensitive to absorption enhancer-induced cytotoxicity compared with the whole intestinal tissue [38,39]. We observed that TEER values significantly reduced by the treatment with Capryol 90 (Figure 8A). Furthermore, Capryol 90 significantly increased the transport of insulin across the Caco-2 cells and the P_app_ value of insulin (Figure 8B). The mechanisms by which Capryol 90 increases the intestinal absorption of insulin are not comprehensively understood. A possible mechanism underlying the improved intestinal insulin absorption mediated by Capryol 90 could be the increased membrane fluidity of the intestinal epithelium, thereby increasing the intestinal absorption of drugs via a transcellular pathway. We previously reported that some enhancers can increase the membrane fluidity of the intestinal epithelium [40,41,42]. Alternatively, it may be plausible that Capryol 90 could loosen TJs of the intestinal epithelium, thereby increasing the intestinal insulin absorption via a paracellular pathway. Indeed, our present studies revealed that Capryol 90 decreased TEER of Caco-2 cell monolayers, and it has been reported that many absorption enhancers decreased TEER values and increased the intestinal transport of drugs [2,43]. González-Mariscal et al. have reported that TJ proteins play an important role in TJ integrity [44]. Reportedly, altered expression levels of TJ proteins and their distribution patterns correlate with the barrier function [45,46]. Therefore, these mechanisms might be related to the absorption-enhancing effect of Capryol 90, improving the intestinal absorption of poorly absorbed drugs, including peptide and protein drugs. Further mechanistic and pharmacokinetic studies are crucial to understanding the mechanisms responsible for the improved intestinal drug absorption mediated by Capryol 90.

## 5. Conclusions

In conclusion, Labrasol^®^ and its related formulations increased intestinal insulin absorption. Notably, Capryol 90’ improved the intestinal absorption of insulin without inducing serious intestinal membrane damage and its absorption-enhancing effect is reversible. In particular, Capryol 90 markedly enhanced insulin absorption under acidic conditions. Additionally, Capryol 90 temporarily reduces the gastrointestinal barrier function. These findings suggest that Capryol 90 may be an excellent and promising absorption enhancer for improving the intestinal absorption of insulin and may show a similar absorption-enhancing effect on the intestinal absorption of other drugs.

## Figures and Tables

**Table 1:** Compositions of Labrasol^®^ and its related formulations.

Product Name	Chemical Definition	Hydrophobic Group
Labrasol^®^	Caprylocaproyl macrogol-8 glycerides	caproic acid (≤2.0%), caprylic acid (50–80%), capric acid (20.0–50.0%), lauric acid (≤3.0%), myristic acid (≤1.0%)
	Caprylocaproyl polyoxyl-8 glycerides	
Capryol 90	Propylene glycol monocaprylate (type ii)	caprylic acid (≥90%), capric acid (≤3.0%), lauric acid (≤3.0%), myristic acid (≤3.0%), palmitic acid (≤1.0%)
Capryol PGMC	Propylene glycol monocaprylate (type i)	caprylic acid (≥99%), capric acid (≤3.0%), lauric acid (≤3.0%)
Lauroglycol 90	Propylene glycol monolaurate (type ii)	caprylic acid (≤0.5%), capric acid (≤2.0%), lauric acid (≥95.0%), myristic acid (≤3.0%), palmitic acid (≤1.0%)
Lauroglycol FCC	Propylene glycol monolaurate (type i)	caprylic acid (≤0.5%), capric acid (≤2.0%), lauric acid (≥95.0%), myristic acid (≤3.0%), palmitic acid (≤1.0%)
Labrafil M 2125 CS	Linoleoyl macrogol-6 glycerides	palmitic acid (4.0–20.0%), stearic acid (≤6.0%),oleic acid (20.0–35.0%), linoleic acid (50.0–65.0%), linolenic acid (≤2.0%), arachidic acid (≤1.0%), eicosenoic acid (≤1.0%)
	Linoleoyl polyoxyl-6 glycerides	
Maisine 35-1	Glycerol monolinoleate	palmitic acid (4.0–20.0%), stearic acid (≤6.0%), oleic acid (10.0–35.0%), linoleic acid (≥50.0%), linolenic acid (≤2.0%), arachidic acid (≤1.0%), eicosenoic acid (≤1.0%)
	Glyceryl monolinoleate	

**Table 2:** Effects of various formulations on the small intestinal absorption of insulin.

Formulation	Concentration (% *v*/*v*)	*AAC*_0__→__4h_ (%⋅min)	*PA*%	Enhancement Ratio
Control	-	636 ± 636	0.10 ± 0.10	-
+ Labrasol^®^	10	5700 ± 130 **	0.87 ± 0.02 **	9.0
+ Capryol 90	5	1040 ± 550	0.16 ± 0.08	1.6
	10	8050 ± 703 **	1.23 ± 0.11 **	12.7
	15	9870 ± 2740 **	1.51 ± 0.42 **	15.5
+ Capryol PGMC	10	2750 ± 1100	0.42 ± 0.17	4.3
+ Lauroglycol 90	10	5700 ± 1350 **	0.87 ± 0.21 **	9.0
+ Lauroglycol FCC	10	844 ± 286	0.13 ± 0.04	1.3
+ Labrafil M 2125 CS	10	10 ± 10	0.002 ± 0.002	0.02
+ Maisine 35-1	10	1110 ± 1100	0.17 ± 0.17	1.7

The plasma glucose levels were measured after intestinal administration of insulin and various formulations and intestinal absorption of insulin (80 IU/kg) was evaluated in these formulations. Each value represents the mean ± S.E. (*n* = 3–4). AAC means area above the curve and PA shows pharmacological availability of insulin, compared with intravenous administration. (**) *p* < 0.01, compared with the control.
